# Diagnostic accuracy and predictive value of the QuantiFERON-TB gold plus assay for tuberculosis in immunocompromised individuals: a prospective TBnet study

**DOI:** 10.1016/j.lanepe.2025.101416

**Published:** 2025-08-06

**Authors:** Martina Sester, Neus Altet-Gomez, Åse Bengaard Andersen, Miguel Arias-Guillén, Korkut Avsar, Anne-Marte Bakken Kran, Graham Bothamley, Anne Christine Nordholm Breschel, James Brown, Dumitru Chesov, Nelly Ciobanu, Daniela Maria Cirillo, Valeriu Crudu, Malu de Souza Galvao, Asli Görek Dilektasli, José Dominguez, Raquel Duarte, Anne Ma Dyrhol-Riise, Delia Goletti, Harald Hoffmann, Elmira Ibraim, Barbara Kalsdorf, Marcin Krawczyk, Heinke Kunst, Berit Lange, Marc Lipman, Alberto Matteelli, Piotr Milkiewicz, David Neyer, Martin Nitschke, Haluk Barbaros Oral, Juan José Palacios-Gutiérrez, Elisa Petruccioli, Joanna Raszeja-Wyszomirska, Pernille Ravn, Jan Rupp, Hanna-Elisa Spohn, Corina Toader, Raquel Villar-Hernandez, Dirk Wagner, Frank van Leth, Leonardo Martinez, Ole Skouvig Pedersen, Christoph Lange

**Affiliations:** a Department of Transplant and Infection Immunology, Saarland University, Homburg, Germany; b Center for Gender-specific Biology and Medicine (CGBM), Saarland University, Homburg, Germany; c Unidad Clinica de Tratamiento. Directamente Observado “Serveis Clinics”, Barcelona, Spain; d Department of Infectious Diseases, Odense University Hospital, Odense, Denmark; e Respiratory Department, Central University Hospital of Asturias, ISPA, Faculty of Medicine, University of Oviedo, CIBER-Respiratory Diseases, Carlos III Health Institute, Oviedo, Spain; f Department of Infectious Diseases, Asklepios Fachklinik München-Gauting, Munich, Germany; g Division of Infection Control, Norwegian Institute of Public Health (NIPH), Oslo, Norway; h Homerton University Hospital, London, UK; i Department of Infectious Disease Epidemiology and Prevention, Statens Serum Institut, Copenhagen, Denmark; j Department of Infectious Diseases, Rigshospitalet, Copenhagen University Hospital, Copenhagen, Denmark; k Department of Respiratory Medicine, Royal Free London NHS Trust and UCL Respiratory, University College London, UK; l Discipline of Pneumology and Allergology Nicolae Testemitanu State University of Medicine and Pharmacy, Republic of Moldova; m Clinical Infectious Disease. Department of Clinical Infectious Diseases, Research Center Borstel, Leibniz Lung Center, Borstel, Germany; n German Center for Infection Research (DZIF), Tuberculosis Unit, Borstel, Germany; o National TB Reference Laboratory, Pneumology Institute, Chisinau, Moldova; p IRCCS San Raffaele Scientific Institute, Milan, Italy; q Pneumology Service, Hospital Universitari Vall d’Hebron, Barcelona, Spain; r Department of Pulmonary Medicine, Bursa Uludag University Faculty of Medicine, Bursa, Turkey; s Servei de Microbiologia, Hospital Universitari Germans Trias i Pujol, Institut d'Investigació Germans Trias i Pujol, Badalona, Spain; t CIBER Enfermedades Respiratorias, Badalona, Spain; u Universidad Autónoma de Barcelona, Barcelona, Spain; v Instituto Nacional De Saúde Dr Ricardo Jorge do Porto, Porto, Portugal; w Instituto de Saúde Pública da Universidade do Porto; Porto, Portugal; x Department of Infectious Diseases, Oslo University Hospital, Oslo, Norway; y Institute of Clinical Medicine, University of Oslo, Oslo, Norway; z Translational Research Unit, National Institute for Infectious Diseases “Lazzaro Spallanzani”, Istituto di Ricovero e Cura a Carattere Scientifico (IRCCS), Rome, Italy; aa Institute of Microbiology and Laboratory Medicine, IML red GmbH; WHO - Supranational Tuberculosis Reference Laboratory Munch-Gauting; Gauting, Germany; ab SYNLAB Gauting, SYNLAB MVZ Dachau GmbH, Munich-Gauting, Germany; ac Department of Clinical Research, Marius Nasta Institute of Pneumophtiziology, Bucharest, Romania; ad Respiratory Medicine & International Health, University of Lübeck, Lübeck, Germany; ae Department of Gastroenterology, Hepatology and Transplant Medicine, Medical Faculty, University of Duisburg-Essen, Essen, Germany; af Laboratory of Metabolic Liver Diseases, Department of General, Transplant and Liver Surgery, Centre for Preclinical Research, Medical University of Warsaw, Warsaw, Poland; ag Queen Mary & Barts Health Tuberculosis Centre, Blizard Institute, Faculty of Medicine & Dentistry, Queen Mary University of London, London, UK; ah Department of Epidemiology, Helmholtz Centre for Infection Research (HZI), Braunschweig, Germany; ai German Center for Infection Research (DZIF), Braunschweig, Germany; aj Clinic of Infectious and Tropical Diseases, Department of Clinical and Experimental Medicine, WHO Collaboration Centre for Tuberculosis Prevention, University of Brescia, Brescia, Italy; ak Department of Hepatology, Transplantology and Internal Medicine, Medical University of Warsaw, Warsaw, Poland; al Translational Medicine Group, Pomeranian Medical University, Szczecin, Poland; am Division of Infectious Diseases, Department of Internal Medicine II, Freiburg University Medical Centre, Freiburg, Germany; an Transplant Center, University Hospital of Schleswig-Holstein, Lübeck, Germany; ao Department of Immunology, Faculty of Medicine, Bursa Uludag University, Bursa, Turkey; ap Regional Mycobacteria Reference Unit, Central University Hospital of Asturias, Instituto de Investigación Sanitaria del Principado de Asturias (ISPA), Oviedo, Spain; aq Section of Infectious Diseases, Department of Medicine, Herlev and Gentofte Hospital, University of Copenhagen, Denmark; ar Infectious Disease Clinic and Institute of Medical Microbiology, University Hospital Schleswig-Holstein, Lübeck, Germany; as German Center for Infection Research (DZIF), Partner Site Hamburg-Lübeck-Borstel-Riems, Lübeck, Germany; at Pathology Department, Marius Nasta Institute of Pneumophysiology, Bucharest, Romania; au Department of Health Sciences, Vrij Universiteit Amsterdam, the Netherlands; av Amsterdam Public Health Research Institute, Amsterdam, the Netherlands; aw Boston University, School of Public Health, Department of Epidemiology, Boston, MA, USA; ax Department of Respiratory Diseases and Allergy, Aarhus University Hospital, Aarhus, Denmark; ay Department of Clinical Medicine, Aarhus University, Aarhus, Denmark; az Global TB Program, Baylor College of Medicine and Texas Children's Hospital, Houston, TX, USA; ba Queen Mary University of London, London, UK; bb London School of Hygiene and Tropical Medicine, London, UK

**Keywords:** IGRA, Immunocompromised individuals, TBnet, Tuberculosis, Progression to tuberculosis

## Abstract

**Background:**

In low tuberculosis (TB)-endemic countries, tuberculosis preventive therapy (TPT) is recommended for immunocompromised individuals with a positive immunodiagnostic test. This study aimed to assess the performance of the QuantiFERON-TB Gold Plus (QFT+) assay and predictive power for future tuberculosis in immunocompromised individuals.

**Methods:**

In this prospective observational study, immunocompromised adults ≥18 years of age including people living with HIV (PLHIV), chronic renal failure, rheumatoid arthritis, solid-organ transplantation or stem-cell transplantation, and immunocompetent adults with and without TB-disease were recruited at 21 sites in 11 European countries and tested with the QFT+ assay. Individuals without TB-disease were followed up for the development of tuberculosis. TB incidence rates (IR) were calculated, stratified by QFT+ results and acceptance of TPT. This study is registered with Clinicaltrials.gov, NCT02639936.

**Findings:**

A total of 2663 individuals (1115 female, 1548 male) were enrolled from 03/11/2015 to 29/03/2019. Persons without tuberculosis were followed up for at least two years. Among 1758 immunocompromised individuals without active tuberculosis, 13.6% had positive QFT+ results. Sensitivity and specificity for TB-disease were 70.0% (52.1–83.3%) and 91.4% (89.6–92.9%), respectively, in immunocompromised, and 81.4% (76.6–85.3%) and 96.0% (92.5–97.9%), respectively, in immunocompetent individuals. During 2457 cumulative years of follow-up among 932 individuals with chronic renal failure, rheumatoid arthritis, solid-organ transplantation or stem-cell transplantation, including 83 persons with a positive QFT+ test without TPT, no-one developed active tuberculosis. In contrast, among 642 PLHIV without TPT, one with an indeterminate QFT+ and 3/30 individuals with a positive QFT+ developed active tuberculosis; all had detectable HIV-replication and low CD4 T-cell counts (incidence 4.1 (95% CI (1.3–12.4) per 100 person-years). No individuals receiving TPT developed active tuberculosis during 269 years of follow-up.

**Interpretation:**

In immunocompromised individuals in low TB-endemic countries, the 2-year-risk for active tuberculosis was highest among PLHIV with detectable HIV-replication and low CD4-counts. In this study, the QFT+ assay did not strongly predict progression to active tuberculosis, which emphasises the need to incorporate additional risk factors.

**Funding:**

None.

**Research in context**

**Evidence before this study**

In the absence of a highly effective vaccine, prevention of tuberculosis in low tuberculosis-incidence countries relies on the identification of individuals with *Mycobacterium tuberculosis* infection, who are at highest risk for progression to active disease. These include close tuberculosis contacts, recent immigrants from high-burden countries, and immunocompromised individuals. *M. tuberculosis* infection is defined indirectly by a reactive immunodiagnostic test, such as a tuberculin-skin-test or an interferon-γ (IFN-γ) release assay (IGRA) in the absence of active tuberculosis. Previous generations of IGRAs had poor predictive value for incident tuberculosis in immunocompromised individuals in countries with low tuberculosis incidence. In 2015, the QuantiFERON-TB Gold in-tube assay, the most frequently used IGRA at that time, was substituted by a novel test generation, the QuantiFERON-TB Gold Plus (QFT+) assay, aiming to improve test performance by simultaneously stimulating *M. tuberculosis*-specific CD4+ and CD8+ T cells *ex vivo* in two different blood tubes. The QFT + assay is now widely available and used to ascertain the risk for progression to tuberculosis in immunocompromised individuals. It has also been used to help diagnose tuberculosis disease, although the test is not generally recommended for this indication. However, its diagnostic accuracy and predictive value in this group of individuals has not been evaluated for either clinical scenario. We searched Pubmed when ethical approval was obtained on the 8th of December 2015 without language restriction for original articles that have used the QFT + assay using the following search strategy (1900/01/01:2015/12/08 [Date–Publication] AND (“QFT-Plus” [All Fields] OR (“QuantiFERON-TB” [All Fields] AND (“gold” [Supplementary Concept] OR “gold” [All Fields] OR “gold” [MeSH Terms]) AND “Plus” [All Fields]) OR (“quantiferontb” [All Fields] AND (“gold” [Supplementary Concept] OR “gold” [All Fields] OR “gold” [MeSH Terms]) AND “Plus” [All Fields])). The search returned no publication neither in immunocompetent nor in immunocompromised persons. We repeated this search on the 26th of May 2025 and found 339 articles. Among those, 14 studies were relevant to the present study as they described the use of the QFT+ assay in individual groups of immunocompromised persons or immunocompetent controls. However, there was no head-to-head study on this assay among several different immunocompromised patient groups and immunocompetent controls, which also included prospective analysis of incident tuberculosis depending on QFT+ test result.

**Added value of this study**

We performed a manufacturer-independent prospective observational cohort study in 21 centers from 11 countries in Europe to ascertain the diagnostic accuracy of the QFT+ assay for the diagnosis of *M. tuberculosis* infection and active tuberculosis in immunocompetent and five groups of immunocompromised individuals, and to evaluate the incidence rates for progression to tuberculosis depending on QFT+ results.

This is the largest head-to-head multicentre study on evaluation of the QFT+ assay in five groups of immunocompromised persons and immunocompetent controls in low-incidence countries, including prospective analysis of incident tuberculosis depending on the QFT+ test result. Overall, the risk of incident tuberculosis was low in immunocompromised individuals in Europe with no individual with chronic renal failure, rheumatoid arthritis, solid-organ transplantation and stem-cell transplantation developing tuberculosis during follow-up. The only immunocompromised individuals who progressed to incident tuberculosis on follow-up were people living with HIV (PLHIV), with the highest incidence among those not using tuberculosis preventive therapy (TPT) and unsuppressed HIV-replicationIn general, the QFT+ assay did not meet the World Health Organization (WHO)-suggested minimal requirement for a non-sputum near point-of-care target product profile assay for the diagnosis of active tuberculosis.

**Implications of all the available evidence**

In low tuberculosis incidence countries, we show that the risk for developing active tuberculosis varies between different immunocompromised groups, with most having a low risk unless there is a clear history of exposure to *M. tuberculosis*. However, the risk is increased in PLHIV with detectable HIV-replication and low CD4-counts, especially when they originate from a country of medium- or high tuberculosis incidence. TPT-strategies in countries of low tuberculosis incidence should prioritise this population at risk. Like the previous test generation, the QuantiFERON-TB Gold in-tube assay, the QFT+ assay poorly predicted incident tuberculosis in immunocompromised individuals in our study.

## Introduction

Tuberculosis is a leading cause of morbidity and mortality worldwide. The World Health Organization (WHO) estimates that 10.8 million people developed tuberculosis in 2023.^1^ This is a historical peak in the number of persons reported by the WHO for this disease worldwide, and is a concern. The only licensed vaccine for the prevention of tuberculosis for more than 100 years is a live attenuated vaccine with *Mycobacterium bovis* bacille Calmette-Guérin (BCG).^2^ The BCG-vaccine, which is administered to almost 90% of newborns world-wide, provides protection from tuberculosis only during the first 5–10 years of life.^3^^,^^4^

In the absence of a more effective vaccine, tuberculosis prevention relies on immunodiagnostic tests to identify individuals with *M. tuberculosis* infection who have the highest risk for progression to active disease,^5^ including close tuberculosis contacts and migrants from high tuberculosis incidence countries. Individuals considered at risk include immunocompromised individuals, such as people living with HIV (PLHIV), recipients of stem-cell transplants (SCT) or solid-organ transplants (SOT), persons with rheumatoid arthritis (RA), and persons with chronic renal failure (CRF).^6^ Some biological agents, especially tumor necrosis factor (TNF)-antagonists and Janus kinase (JAK) inhibitors lead to a substantial risk of tuberculosis reactivation in individuals with *M. tuberculosis* infection.^7^ Following implementation of screening policies in individuals with autoimmune disease, the incidence of tuberculosis could be substantially reduced in this population.^8^ The most widely used immunological assays that are used as immunodiagnostic tests for *M. tuberculosis* infection are the tuberculin skin-test (TST) and interferon-γ release assays (IGRAs), for which two commercial tests are mainly in use, i.e., the ELISPOT-based T. SPOT.*TB* and the ELISA-based QuantiFERON-TB (QFT) assay. In general, IGRAs demonstrate superior performance for predicting incident tuberculosis compared with the TST.^9^^,^^10^ Nevertheless, we have previously shown in PLHIV that the TST was not inferior to IGRAs in predicting the development of active tuberculosis.^5^ Moreover, both the IGRAs and TST have higher predictive value in low versus high endemic settings.^11^ Both tests lack specificity for active tuberculosis as they are unable to discriminate individuals who are latently infected from those with active disease. Thus the tests do not meet the World Health Organization (WHO)-suggested minimal requirement for a non-sputum near point-of-care target product profile assay for the diagnosis of active tuberculosis.^5^^,^^12^, ^13^, ^14^ In addition, quantitative IGRA responses do not distinguish between remote infection and recent infection with *M. tuberculosis*, where the progression to active tuberculosis is highest.^15^

As a result of a prospective study across 17 centers in 11 European countries, the Tuberculosis Network European Trialsgroup (TBnet; www.tbnet.eu) reported in 2014 that the TST, the T-SPOT. *TB* test, and the QuantiFERON-TB Gold in-Tube test (QFT) vary substantially among different groups of individuals with immunodeficiencies, and are poor predictors for the development of tuberculosis in immunocompromised individuals.^16^

In 2015, a new version of the QFT became available, the QuantiFERON-TB Gold Plus assay (QFT+). QFT+ uses two different tubes with *M. tuberculosis*-specific antigens, the TB1-tube with a cocktail of peptides derived from ESAT-6 and CFP-10 primarily stimulating CD4 T cells, and the TB2-tube containing peptides from TB1 plus additional short peptides from CFP-10 optimised to stimulate CD8 T cells, thereby designed to elicit both CD4 and CD8 T cells.^17^, ^18^, ^19^ In a systematic review and meta-analysis, the QFT+ test was found to be more sensitive compared to QFT for detecting *M. tuberculosis* infection, mainly due to TB2 responses.^20^ However differences in TB1 and TB2 for the diagnosis of tuberculosis in immunocompromised individuals, or the ability of this test for prediction of the development of tuberculosis were not addressed.^20^ In a study from China where QFT+ responses in TB1 and TB2 were analyzed as indicators for tuberculosis recurrence, individuals with a TB1−/TB2+ status exhibited stronger association with the risk of tuberculosis recurrence than those with a TB1+/TB2+ status indicating a possible benefit of the additional tube.^21^ A limitation of the QFT+ test is substantially diminished IFN-γ responses among PLHIV, particularly those with lower CD4 counts as well as persons with diabetes mellitus.^22^

This study has two main objectives towards providing evidence for immunodiagnostic testing to guide TPT in both immunocompetent and immunocompromised individuals in low-incidence European countries: a) To evaluate the sensitivity and specificity of the QFT+ test among immunocompetent and immunocompromised individuals with active tuberculosis and without risk factors for exposure, and whether the addition of the TB2 tube provides a benefit for identification of *M. tuberculosis* infected individuals, and b) to prospectively evaluate the incidence rates for progression to tuberculosis depending on QFT+ results.

## Methods

### Study design and aims

**Fig. 1:**
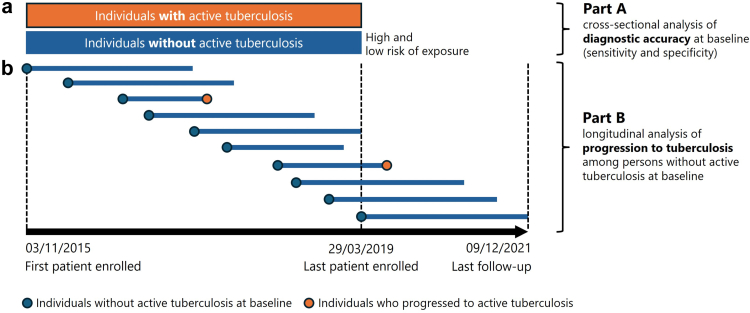
**Study design including timeline of patient enrollment for baseline analyses and follow-up for incident tuberculosis. (a)** In the cross-sectional part of the study, all participants underwent QFT+ testing at individual sites. QFT+ results, along with demographic and clinical characteristics including risk factors for *Mycobacterium tuberculosis* exposure were collected using a standardised questionnaire. Active tuberculosis at the time of QFT+ testing was defined by nucleic acid amplification test positivity and/or culture confirmation. The diagnostic accuracy of QFT+ for detecting active tuberculosis at baseline (sensitivity and specificity) was assessed in this part of the study (part A). Tuberculosis preventive therapy (TPT) was administered according to individual site practice, at the discretion of the treating physicians. **(b)** Part B of the study prospectively assessed the progression to incident active tuberculosis, stratified by baseline QFT+ result. All individuals without tuberculosis at baseline were subsequently followed for at least two years after enrolment or until the development of incident tuberculosis or censoring (date when patients had their last clinical assessment, died, or at the end of study, whichever occurred first). Horizontal lines represent examples of individual persons with variable observation times for follow-up. The median duration of follow-up was 2.6 (interquartile range 2.1–3.4) years.

In this prospective observational study, individuals from five different groups of immunocompromised persons were recruited along with immunocompetent controls. Study participants were enrolled in 21 TBnet study centers (specialised centers for the treatment of individuals with tuberculosis and/or persons with immunodeficiencies) across Europe, tested with the QFT+, and followed for the development of active tuberculosis. The study was divided in two parts with a schematic representation of the study design and the two primary aims shown in Fig. 1a and b.

### Recruitment of study population

Study participants were recruited from 21 TBnet centers across Europe. Inclusion criteria for the cross-sectional part of the study were adults ≥18 years of age with a diagnosis of HIV-infection, chronic renal failure on renal replacement therapy, rheumatoid arthritis, solid-organ transplantation (lung, liver, kidney, kidney-pancreas), or stem-cell transplantation. Immunocompetent individuals with and without risk factors for exposure were included as controls and were recruited by the same centers (including staff from hospital, research institutes or administrative departments). Both immunocompetent and immunocompromised individuals with and without active tuberculosis were included. Diagnosis of active tuberculosis at the time of testing was based on nucleic acid amplification test positivity and/or culture confirmed tuberculosis. Study participants were consecutively identified (enrolment depended on participants’ consents and eligibility criteria) and recruited from 03/11/2015 to 29/03/2019 during routine care. TPT was given according to individual center practice at the discretion of the treating physicians. Inclusion criteria for the prospective part of the study were all individuals without active tuberculosis who participated in the cross-sectional study. Regardless of TPT, all individuals were followed for the development of tuberculosis for at least 2 years with last follow-up on 09/12/2021 (Fig. 1b).

The study was approved by the ethics committee of the Physicians‘ council of the Saarland Federal State of Germany (Ethikkommission der Ärztekammer des Saarlandes, reference 221/15, approval date 8th of December 2015), and all local ethics committees, and all participants gave written informed consent.

### Study design and data collection

In a cross-sectional part of the study, all study participants underwent QFT+ testing at the individual sites. QFT+ results as well as demographic and clinical characteristics including risk factors for *M. tuberculosis* exposure were collected at the sites using a standardised questionnaire. Data regarding sex (male, female) and ethnicity (Caucasian (white), Hispanic, African/Afro-American (black), Asian) were collected by the respective staff in each study center. Data were electronically transmitted to the coordinating center at Saarland University. All individuals without active tuberculosis entered a prospective part of the study, where follow-up information on the occurrence of active tuberculosis after initial testing as well as information on TPT was collected by the treating physician (Fig. 1). The study was reported in accordance with the *Standards for Reporting Diagnostic Accuracy Studies* (Supplementary Table S1).

### Technical procedures, data sources and exposure variables

In both the cross-sectional part of the study, which assessed the diagnostic accuracy of the QFT+ assay, and the prospective part of the study, which evaluated the progression from tuberculosis infection to active tuberculosis, the QFT+ assay served as the index test. The test was performed and interpreted according to the manufacturer's instructions (Qiagen, Hilden, Germany) with details given in the Supplementary Methods. The laboratory personnel conducting the testing were blinded to the clinical status of the study participants. For the cross-sectional part of the study, tuberculosis at the time of QFT+ testing was based on nucleic acid amplification test positivity and/or culture confirmation, consistent with the gold standard for active tuberculosis diagnostics. In the prospective part of the study, the reference standard was microbiologically confirmed incident tuberculosis. As in our previous study,^16^ incident tuberculosis was defined as tuberculosis occurring more than 30 days after QFT+ testing to avoid misclassification of prevalent disease as incident cases. “*M. tuberculosis* exposure” was defined as previously described^16^ and included a reported history of known exposure to *M. tuberculosis*, active tuberculosis, tuberculosis treatment, *M. tuberculosis* infection, TPT for *M. tuberculosis* infection, or being a resident in a high tuberculosis incidence country for at least 1 year. Individuals with or without these risk factors were classified as high-risk or low-risk, respectively.

### Statistical analysis

Categorical variables were presented as absolute numbers with proportions, continuous variables were reported as medians with interquartile ranges. In the cross-sectional part of the study, the crude diagnostic accuracy of the QFT+ was assessed by calculating sensitivity (proportion of positive QFT+ among all individuals with tuberculosis) and specificity (proportion of negative QFT+ among all individuals without risk factors) for identifying active tuberculosis at baseline in both immunocompromised and immunocompetent individuals, using microbiologically confirmed tuberculosis as the reference standard. Exact 95% confidence intervals were calculated based on the binomial distribution. In addition, to account for heterogeneity between countries, country-specific and pooled sensitivities and specificities were calculated using random-effects meta-analyses of proportions with logit transformations (using *metaprop* from the R package *meta*).^23^ Weights were assigned to each country using the inverse variance method. Between-country variability (τ^2^) was estimated using the DerSimonian–Laird method, and heterogeneity was quantified using the I^2^ statistic. Confidence intervals for individual countries were calculated using the Clopper–Pearson method. Knapp–Hartung adjustments were applied to the random-effects model. Proportions were presented on the original scale (%).^24^

To compare the performance of TB1 and TB2 in different groups, frequency of test positivity was calculated for TB1 and TB2. Differences in the percentage of positive tests between TB1 and TB2 were assessed using McNemar's test based on exact binomial probabilities.^25^^,^^26^ In a separate analysis, the percentages of indeterminate results were compared across the same groups.

In the prospective part of the study, risk time was calculated as years from the date of testing to the date when patients had their last clinical assessment, developed active tuberculosis, died, or at the end of study, whichever occurred first. As active tuberculosis diagnosed within 30 days following testing was considered as co-prevalent tuberculosis rather than a valid case of incident tuberculosis, only individuals with more than 30 days of follow-up were included in the prospective analysis. To mitigate immortal time bias in subsequent analyses, the risk period was recalculated by subtracting 30 days from the individual endpoint.

Incidence rates (IR) of tuberculosis were calculated for immunocompromised and immunocompetent individuals, stratified by QFT+ results and acceptance of TPT. IR of tuberculosis were also calculated for PLHIV stratified by QFT+ results and HIV-load. To compare subgroups, incidence rate ratios (IRR) were calculated using immunocompetent individuals with negative QFT+ results without TPT as the reference group. Time to progression to tuberculosis was assessed using Aalen-Johansen curves for the whole cohort and subgroups, considering death as a competing risk. Differences between subgroups were assessed using Gray's test for equivalence of cumulative incidence functions.

Regarding handling of missing data, individuals where QFT+ results could not be obtained due to technical or logistical failure were excluded prior to analysis. All other QFT+ results (positive, negative, indeterminate) were included in the cross-sectional analysis of diagnostic accuracy. In the prospective analysis of patients without active tuberculosis at baseline, subgroup analyses involving HIV load and CD4 counts were conducted as complete-case analyses, including only those participants with available data on these variables.

All statistical analyses were performed in R v. 4.3.2. Graphics were prepared using R and GraphPad Prism (10.0.3) for Windows (Boston, Massachusetts USA).

The study was registered under ClinicalTrials.gov NCT02639936.

### Role of the funding source

There was no funding source for this study.

## Results

### Study population

**Fig. 2:**
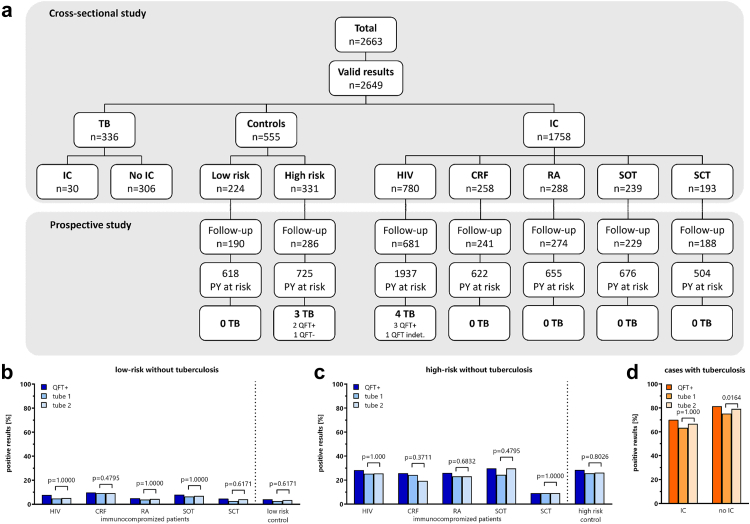
**Flow chart of immunocompromised individuals and controls recruited into the study and cases of active tuberculosis during follow-up and results of immune-based testing. (a)** The number of individuals analysed at baseline in the cross-sectional study, and the number of individuals who were included to assess development of tuberculosis on follow-up (incident cases). All individuals with more than 30 days of follow-up were included, with the number of individuals and cumulative person-years of follow-up indicated for each group. HIV, human immunodeficiency virus infection. The percentage of positive QFT+ test-results among immunocompromised individuals and immunocompetent controls without tuberculosis **(b)** without and **(c)** with risk-factors for *M. tuberculosis*, and in **(d)** immunocompromised individuals and immunocompetent controls with tuberculosis was quantified. Qualitative results (positive and negative) are shown for the QFT+ assay as well as separately for tube 1 and tube 2; statistical analysis was carried out using McNemar's test for matched samples; percentages of positive, negative and indeterminate test results for each group are given in Table 1. HIV, human immunodeficiency virus infection; CRF, chronic renal failure; IC, immunocompromised; PY, person-year; RA, rheumatoid arthritis; SOT, solid-organ transplantation; SCT, stem-cell transplantation; TB, tuberculosis.

A total of 2663 individuals were enrolled at 21 sites in 11 European countries within the TBnet consortium (Denmark, Germany, Italy, Republic of Moldova, Norway, Poland, Portugal, Romania, Spain, Turkey, and UK), of which 2649 had evaluable QFT+ results (861 immunocompetent controls and 1788 individuals with immunodeficiency). Among 1758 immunocompromised individuals without active tuberculosis, causes of immunodeficiency included HIV-infection (44.4%, n = 780), CRF (14.7%, n = 258), RA (16.4%, n = 288), SOT (13.6%, n = 239), and SCT (11.0%, n = 193, Fig. 2a and Table 1). Moreover, 30 immunocompromised patients were included who had active tuberculosis at the time of testing (26 PLHIV, 2 RA and 2 CRF). In addition, 555 immunocompetent persons without tuberculosis (224 low-risk and 331 high-risk controls), and 306 immunocompetent patients with tuberculosis were enrolled (Fig. 2a and Table 1). Immunosuppressive and/or immunomodulatory drug regimens in persons with RA and in SOT or SCT recipients are shown in Supplementary Tables S2 and S3, respectively. The prospective study included 476 controls and 1613 immunocompromised individuals without active tuberculosis with a cumulative follow-up time of 5737 years (median 2.6 (IQR 2.1–3.4) years, Fig. 2a).

**Table 1:** Demographic characteristics of controls and immunocompromised persons stratified by type of immunosuppression.

	HIV	CRF	RA	SOT	SCT	IC persons with TB^a^	Low-risk Controls	High-risk Controls	Controls with TB
Total, n	792	258	290	239	193	30	224	331	306
Male, n (%)	557 (70.3%)	165 (64.0%)	83 (28.6%)	137 (57.3%)	113 (58.5%)	23 (76.7%)	116 (50.7%)	128 (39.3%)	226 (73.9%)
Female, n (%)	235 (29.7%)	93 (36.0%)	207 (71.4%)	102 (42.7%)	80 (41.5%)	7 (23.3%)	113 (49.3%)	198 (60.7%)	80 (26.1%)
Age (median, IQR)	54.4 (47.1–82.1)	67.7 (57.1–78.1)	56.9 (47.7–66.5)	55.0 (45.2–63.3)	59.1 (49.2–66.8)	42.9 (32.2–52.0)	28.6 (22.0–48.9)	44.0 (32.3–56.1)	36.9 (26.1–50.1)
Race and ethnicity, n (%)									
Asian	48 (6.1%)	9 (3.5%)	3 (1.0%)	6 (2.5%)	3 (1.6%)	1 (3.3%)	0 (0%)	11 (3.4%)	53 (17.3%)
Black	132 (16.7%)	6 (2.3%)	4 (1.4%)	3 (1.3%)	0 (0%)	6 (20.0%)	0 (0%)	7 (2.1%)	76 (24.8%)
Hispanic	27 (3.4%)	0 (0%)	0 (0%)	1 (0.42%)	1 (0.52%)	2 (6.7%)	1 (0.45%)	10 (3.1%)	6 (2.0%)
White	577 (72.9%)	241 (93.4%)	276 (95.2%)	216 (90.4%)	181 (93.8%)	18 (60.0%)	228 (99.6%)	298 (91.4%)	168 (54.9%)
Unknown	8 (1.0%)	2 (0.8%)	7 (2.4%)	13 (5.4%)	8 (4.1%)	3 (10.0%)	0 (0%)	0 (0%)	3 (1.0%)
Immigrants, n (%)	277 (35.0%)	40 (15.5%)	28 (9.7%)	23 (9.6%)	28 (14.5%)	16 (53.3%)	3 (1.3%)	58 (17.8%)	187 (61.1%)
Years since immigration (median, IQR)	18.0 (12.8–49.1)	29.5 (21.4–41.4)	33.2 (23.6–46.0)	26 (18.5–31.3)	22.7 (18.8–28.4)	5.9 (0.7–8.8)	21.2 (9.3–26.1)	11.7 (7.5–21.9)	2.3 (0.8–7.0)
Risk factors for *M. tuberculosis* exposure^b^, n (%)	344 (43.3%)	62 (24.0%)	110 (37.9%)	37 (15.5%)	44 (22.8%)	30 (100%)	0 (0%)	331 (100%)	306 (100%)
History of exposure to *M. tuberculosis*	40 (5.1%)	27 (10.5%)	34 (11.7%)	18 (7.5%)	25 (13%)	5 (16.7%)	0 (0%)	134 (41.1%)	83 (27.1%)
History of active TB	38 (4.8%)	8 (3.1%)	5 (1.7%)	7 (2.9%)	3 (1.6%)	7 (23.3%)	0 (0%)	11 (3.4%)	40 (13.1%)
History of tuberculosis treatment	34 (4.3%)	10 (3.9%)	7 (2.4%)	3 (1.3%)	1 (0.5%)	8 (26.7%)	0 (0%)	7 (2.1%)	37 (12.1%)
History of *M. tuberculosis* infection	39 (4.9%)	7 (2.7%)	72 (24.8%)	5 (2.1%)	6 (3.1%)	6 (20.0%)	0 (0%)	70 (21.1%)	22 (7.2%)
History of TPT	17 (2.1%)	2 (0.8%)	41 (14%)	2 (0.8%)	2 (1.0%)	1 (3.3%)	0 (0%)	20 (6.0%)	3 (1.0%)
>1 year in high TB-incidence country	313 (39.5%)	38 (14.7%)	36 (14.7%)	14 (5.9%)	19 (9.8%)	25 (83.3%)	0 (0%)	129 (39.6%)	234 (76.5%)
No known risk factors for *M. tuberculosis* exposure^b^, n (%)	448 (56.6%)	196 (76.0%)	180 (62.1%)	202 (84.5%)	149 (77.2%)	0 (0%)	224 (100%)	0 (0%)	0 (0%)
Tests performed^c^	780 (98.5%)	258 (100%)	288 (99.3%)	239 (100%)	193 (100%)	30 (100%)	224 (100%)	331 (100%)	306 (100%)
Indeterminate results	5 (0.6%)	5 (1.9%)	9 (3.1%)	6 (2.5%)	3 (1.6%)	1 (3.3%)	0 (0%)	9 (2.7%)	6 (2.0%)
Positive results	129 (16.5%)	35 (13.6%)	37 (12.8%)	27 (11.3%)	11 (5.7%)	21 (70.0%)	9 (4.0%)	94 (28.4%)	249 (81.4%)
Negative results	646 (82.8%)	218 (84.5%)	242 (84.0%)	206 (86.2%)	179 (92.8%)	8 (26.7%)	215 (96.0%)	228 (68.9%)	51 (16.7%)

a Including 26 HIV-patients, 2 CRF patients, 2 RA patients.

b Refers to self-reported evidence of prior “*M. tuberculosis* exposure” defined by a reported history of either exposure to *M. tuberculosis*, active tuberculosis, tuberculosis treatment, *M. tuberculosis* infection or TPT, or being at least for 1 year a resident in a high TB-incidence country; immigrants were only included in the “*M. tuberculosis* exposure group” if patients had lived in high TB prevalence countries for more than 1 year prior to immigration.

c 14 tests failed based on technical or logistical reasons; HIV, HIV-infection; CRF, chronic renal failure; IC, immunocompromised; RA, rheumatoid arthritis; SOT, solid-organ transplantation; SCT, stem-cell transplantation; TPT, tuberculosis preventive therapy; IQR, interquartile range.

There were differences in demographic characteristics between the immunocompromised groups as well as control groups. All control groups were younger (median 27.0 (IQR 21.9–53.0) years for low-risk, 43.8 (IQR 32.0–56.1) years for high-risk, 36.9 (IQR 26.1–50.1) years for controls with tuberculosis) than immunocompromised groups (all a median of >45 years, Table 1). The highest percentage of persons with a history of immigration was found among controls with tuberculosis (61.1%) and time since immigration was shortest among controls with active tuberculosis (median 2.3 years (IQR 0.8–7.0) ago). Among immunocompromised groups, the highest percentage of individuals with risk factors for *M. tuberculosis* exposure was found for PLHIV (43.3%) with the other groups ranging from 15.5 to 37.9% (Table 1).

### Cross-sectional analysis: QFT+ test results in immunocompetent and immunocompromised individuals at baseline

After exclusion of 14 invalid tests with technical or logistical failures, baseline QFT+ results were available from cross-sectional analysis of 2649 study participants (Fig. 2a). The percentage of indeterminate results was low in controls and all groups of immunocompromised individuals without active tuberculosis (from 0.64% in PLHIV to 3.1% in RA, Table 1) and participants with tuberculosis (3.3% in immunocompromised individuals and 2.0% in immunocompetent controls, Table 1). Overall, the percentage of positive QFT+ results among immunocompromised persons without tuberculosis was 13.6%, ranging from 5.7% in stem-cell transplant recipients to 16.5% in PLHIV (Table 1). Among immunocompetent controls with and without risk factors for exposure, 28.4% and 4.0%, respectively, had a positive QFT+ test (Table 1 and Fig. 2b and c). Immunocompromised individuals without known risk-factors had similarly low percentages of positive QFT+ tests as controls (Fig. 2b). Moreover, except for stem-cell transplant recipients, the percentage of positive QFT+ tests among immunocompromised individuals with high-risk was in the same range as in high-risk controls (Fig. 2c). The highest percentage of positive QFT+ results was observed among patients with tuberculosis with 70.0% in immunocompromised and 81.4% in immunocompetent individuals (Fig. 2d). When comparing test results based on positivity in tube TB1 and tube TB2, there was no difference between the tubes, except for immunocompetent patients with tuberculosis, who had a higher percentage of positive tests in tube TB2 (Fig. 2d).

### Diagnostic accuracy of the QFT+ test among immunocompromised and immunocompetent participants (aim 1)

The sensitivity of the QFT+ test for detecting active tuberculosis was calculated from 30 immunocompromised and 306 immunocompetent participants with tuberculosis, while specificity was calculated in persons without a history of *M. tuberculosis* exposure (224 low-risk controls and 1171 immunocompromised individuals without known risk factors for exposure, Supplementary Table S4 and Fig. 2b and d). Among 530 immunocompetent participants, the observed crude sensitivity and specificity were 81.4% (95% CI 76.6–85.3%) and 96.0% (95% CI 92.5–97.9%), respectively. Among 1201 immunocompromised participants, 21/30 patients with active tuberculosis had a positive QFT+ result, corresponding to a crude sensitivity of 70.0% (95% CI 52.1–83.3%)). Among 1171 participants without risk factors, 1070 had a negative QFT+ result, corresponding to a crude specificity of 91.4% (95% CI 89.6–92.9%).

Random-effects meta-analyses accounting for between-country heterogeneity yielded slightly lower estimates of sensitivity and specificity: In immunocompetent patients, the pooled sensitivity was 76.8% (95% CI 64.6–85.8%) with moderate heterogeneity (I^2^ = 62.7%), and the pooled specificity was 95.0% (95% CI 82.9–98.7%; I^2^ = 49.3%, Supplementary Figure S1a and b). Among immunocompromised patients, the pooled sensitivity was 64.9% (95% CI 42.4–82.3%; I^2^ = 11.3%) and the pooled specificity was 91.2% (95% CI 89.4–92.7%; I^2^ = 0.0%, Supplementary Figure S2a and b), indicating a lower heterogeneity across countries for immunocompromised individuals.

### Prospective study: progression to tuberculosis during follow-up

During follow-up, we observed four co-prevalent cases with tuberculosis within 30 days (two PLHIV after 8 and 29 days, and two controls after 13 and 30 days). Beyond 30 days of follow-up using data from 476 controls and 1613 immunocompromised persons without tuberculosis, seven study participants (0.3%) developed incident tuberculosis (Fig. 2a and Table 2). All had risk factors for prior *M. tuberculosis* exposure. Three of seven progressors were immunocompetent controls. Among immunocompromised progressors, all were PLHIV. Progressors among PLHIV generally had low CD4-counts (<200 cells/μl in 3 of the 4 patients, and one with 360 cells/μl), and all had either detectable blood HIV-copies or were not on antiretroviral therapy (ART). In general, the majority of progressors were immigrants originating from countries with a TB-prevalence of >45/100.000 (85.7%) and most had male sex (71.4%). Five out of seven individuals had a positive QFT+ result at the time of screening, one PLHIV had an indeterminate result, and one control had a negative test result. Six of the seven progressors had not received TPT, whereas its use was unknown for one PLHIV. Among the seven progressors, IFN-γ secretion levels in tube 1 and tube 2 were similar with no meaningful difference between the two tubes (Table 2). Their median IFN-γ levels were 1.3 (IQR 0.8–1.7) IU/mL for TB1 and 1.6 (IQR 1.2–1.7) IU/mL for TB2 (n = 6, one person with an indeterminate test result excluded). These levels were numerically higher as compared to individuals without TPT who did not progress (n = 1937, 0.1 (IQR 0.04–0.2) IU/mL for TB1, and 0.2 (IQR 0.03–0.2) IU/mL for TB2).

**Table 2:** Characteristics of patients developing active tuberculosis during follow-up.

group	Country of birth/actual residence	sex	TB (months after QFT+ testing)	TB type	TB risk	QFT+ result	TB1-Nil (IU/ml)	TB2-Nil (IU/ml)	TB preventive therapy		HIV CDC	ART	HIV load	CD4 T cells/μl
									offered	done				
HIV	Colombia Italy	male	33	both	High/IC	Positive	0.93	1.56	unknown	unknown	A2	Yes (>1 year)	146	360
HIV	Brazil Italy	male	3	extrapul-monary	High/IC	Positive	1.41	1.66	No	no	A3	No	184.230	128
HIV	Moldova Moldova	male	32	pulmonary	High/IC	indeterminate	0.03	0.04	No	no	C3	No	980.352	3
HIV	Brazil Italy	male	19	both	High/IC	Positive	1.46	1.28	No	no	A3	No	120.808	107
Control	UK UK	male	3	extrapul-monary	High	Positive	0.64	0.99	No	no	n.a.	n.a.	n.a.	n.a.
Control	Moldova Moldova	female	28	pulmonary	High	Negative	0	0	No	no	n.a.	n.a.	n.a.	n.a.
Control	Romania Romania	female	7	extrapul-monary	High	Positive	>10	>10	No	no	n.a.	n.a.	n.a.	n.a.

No patient had a history of earlier tuberculosis; ART, antiretroviral therapy; TB, tuberculosis; IS, immunocompromised.

### Incidence of tuberculosis depending on QFT+ result and TPT (aim 2)

The incidence of tuberculosis by QFT+ result and use of TPT is shown in Table 3. In the absence of TPT, the incidence of tuberculosis was higher among people with positive QFT+ results, regardless of immunodeficiency: Among immunocompetent controls who did not receive TPT, tuberculosis-incidence was 0.09 (95% CI 0.01–0.7) and 1.3 (95% CI 0.3–5.4) cases per 100 person-years in individuals with negative and positive QFT+ results, respectively. The incidence was significantly higher in the group with positive QFT+ results, with an incidence rate ratio (IRR) of 14.4 (95% CI 1.3–159.0). Among immunocompromised individuals who did not receive TPT, the incidence of tuberculosis was 0 in those with negative QFT+ results, and 0.6 (95% CI 0.2–2.0) and 1.7 (95% CI 0.2–12.2) cases per 100 person-years in persons with positive and indeterminate QFT+ results, respectively. In comparison to immunocompetent controls with negative QFT+ results, tuberculosis incidence was significantly higher in immunocompromised individuals with indeterminate QFT+ results (IRR 18.6 (95% CI 1.2–297.0)), whereas the difference did not reach statistical significance for immunocompromised individuals with positive QFT+ results (IRR 7.0 (95% CI 0.7–66.9), Table 3). As shown in Table 3, there are 6 controls and 38 immunocompromised persons who received TPT despite negative results. In general, no cases of active tuberculosis occurred during 260 years of cumulative follow-up among the 109 study participants who received TPT. Moreover, none of the immunocompromised individuals with negative QFT+ results developed active tuberculosis, regardless of whether they used TPT (Table 3).

**Table 3:** Incidence of tuberculosis stratified by QFT+ result and use of TB preventive therapy after QFT+ testing for *M. tuberculosis* infection.

	Test-result^b^	TB preventive therapy^c^	n	PY at risk	TB cases	Incidence^a^	Incidence rate ratio^d^
Controls	negative	no	372	1075	1	0.09 (0.01–0.7)	Reference
	negative	yes	6	14	0	0	–
	indeterminate	no	9	24	0	0	–
	indeterminate	yes	0	0	0	0	–
	positive	no	56	149	2	1.3 (0.3–5.4)	14.4 (1.3–159.0)
	positive	yes	33	80	0	0	–
Immunocompromised individuals	negative	no	1334	3700	0	0	–
	negative	yes	38	86	0	0	–
	indeterminate	no	25	58	1	1.7 (0.2–12.2)	18.6 (1.2–297.0)
	indeterminate	yes	3	6	0	0	–
	positive	no	181	464	3	0.6 (0.2–2.0)	7.0 (0.7–66.9)
	positive	yes	32	80	0	0	–

a Incidence is given per 100 person-years (PY); the rates refer to the cumulative rates after testing for *M. tuberculosis* infection.

b This analysis includes all individuals with valid test results (positive, negative, indeterminate).

c TB preventive therapy refers to treatment administered after the QFT+ test performed in this study.

d The reference groups comprised immunocompetent individuals with negative QFT+ results without preventive therapy; Abbreviations: PY, person-years; TB, tuberculosis; TPT, TB preventive therapy.

When restricted to PLHIV not receiving TPT, individuals with positive QFT+ results and detectable HIV-load had an incidence of 4.1 (95% CI 1.3–12.4) per 100 person-years. PLHIV with indeterminate QFT+ results and detectable HIV-load had the highest incidence of tuberculosis (20.0 (95% CI 2.8–142.0) per 100 person-years), although this analysis is limited by low overall numbers in this group (Supplementary Table S5). When compared to immunocompetent individuals with negative QFT+ results who did not receive TPT, this corresponds to an IRR of 44.3 (95% CI 4.6–426.0) for PLHIV with positive, and 216.0 (95% CI 13.5–3446.0) for PLHIV with indeterminate QFT+ results, respectively.

**Fig. 3:**
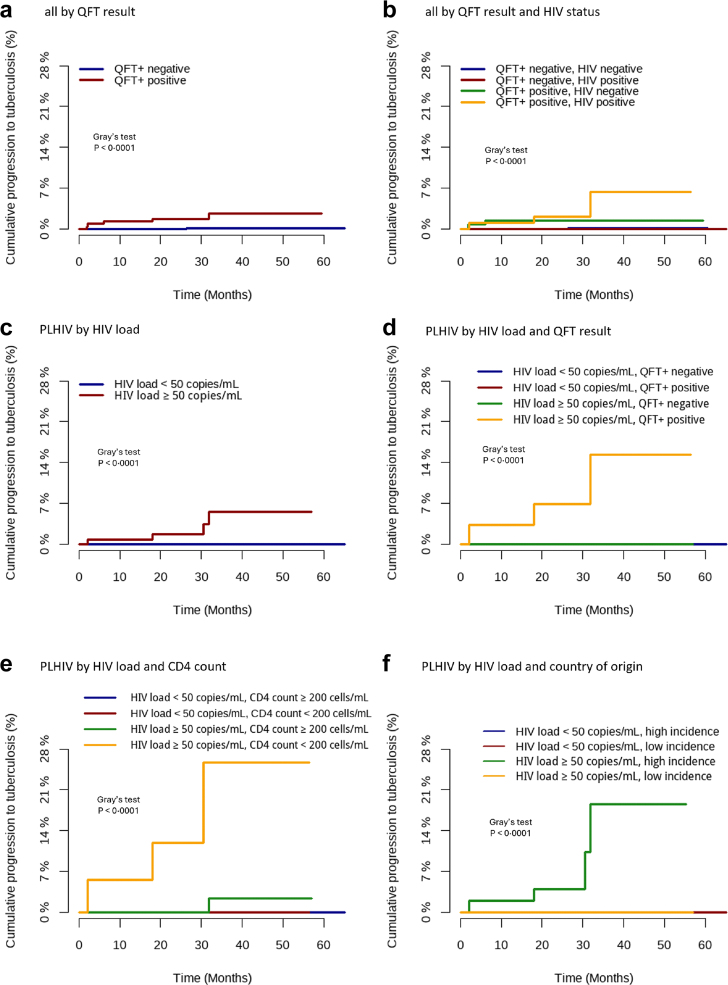
**Time to progression to tuberculosis**. Shown are all participants without tuberculosis preventive therapy (TPT) stratified according to **(a)** QFT+ results, **(b)** QFT+ results and HIV-status. PLHIV were stratified by **(c)** HIV-load, **(d)** HIV-load and QFT+ result, **(e)** HIV-load and CD4-counts, or **(f)** HIV-load and tuberculosis incidence in the country of origin; this analysis was restricted to individuals with positive or negative QFT+ results; the curves were based on the Aalen-Johansen estimator considering death as a competing risk; Comparisons between groups were carried out using Gray's test for equivalence of cumulative incidence function; numbers of individuals at risk are summarised in Supplementary Table S6.

The cumulative incidence of tuberculosis in individuals without TPT stratified by QFT+ result and HIV-status is shown in Fig. 3a and b. Among PLHIV, the cumulative incidence of tuberculosis was significantly higher in those with detectable HIV-load, indicating either a lack of ART use or an ineffective regimen (Fig. 3c). Among PLHIV with detectable HIV-load, the incidence was higher in persons with low CD4 T-cell counts, positive QFT+ tests, or in persons originating from high tuberculosis incidence countries (Fig. 3d–f).

## Discussion

Although immunocompromised individuals are considered at increased risk for the development of active tuberculosis,^6^ their risk in low incidence settings primarily results from the reactivation of a latent infection acquired prior to immunosuppression.^27^ In such contexts, ongoing transmission and new exposure to *M. tuberculosis* are uncommon, which contrasts to high incidence areas, where recent exposure plays a larger role. In our prospective study cohort of more than 1750 adult immunocompromised individuals from 21 sites in 11 European countries, we found that the incidence of tuberculosis was low even in persons not receiving TPT. Surprisingly, incident tuberculosis was not increased in immunocompromised persons compared to immunocompetent controls, even when they originated from high incidence countries. It is noteworthy that incident tuberculosis exclusively occurred among PLHIV, of whom all had additional risk factors for exposure, and ongoing HIV-replication and low CD4-counts as indicators of advanced HIV-infection and marked immunosuppression.

The percentage of *M. tuberculosis* infected individuals identified by QFT+ was 13.6% in all immunocompromised persons and varied from 18.2% in PLHIV to as low as 5.7% in SCT recipients, which is in the range of positivity among low-risk controls without *M. tuberculosi*s exposure. Of note, in all immunocompromised groups except SCT recipients, the percentage of positive QFT+ results was highest among those with risk factors for exposure, and similarly high as in immunocompetent controls with risk factors. Thus, although the sensitivity of IGRAs is known to be affected by the level of immunodeficiency,^28^ our data may indicate that test positivity is more strongly affected by exposure than by the status of immunodeficiency. Test results of the TB1-tube may be considered as a surrogate for the previous QFT version, the QuantiFERON-TB Gold in-tube assay. The addition of the TB2-tube of the QFT+ test provided significant but only marginal benefit for the diagnosis of active tuberculosis in immunocompetent individuals, but still one in five patients with tuberculosis was not identified by the QFT+ test. In immunocompromised individuals the addition of the TB2-tube did not provide any benefit compared to the TB1-tube alone for the diagnosis of active disease or the prediction of tuberculosis, confirming previous findings that found similar performance of the QFT+ and the previous QuantiFERON version.^29^, ^30^, ^31^, ^32^, ^33^, ^34^ A recent study comparing the previous QuantiFERON version with the QFT+ assay found a high level of concordance among patients on long-term immunosuppressive therapy (92%, 115/125). Although 9 patients were identified as positive in the QFT+ assay only, only 4 out of 9 samples had higher IFN-γ levels in the TB2 tube. Thus, despite a potential benefit of the QFT+ test in general, there was no clear benefit of TB2 over TB1.^35^ Moreover, we confirmed that the QFT+ test has a poor diagnostic accuracy for active tuberculosis and that the test should not be applied in this context. This emphasises that the QFT+ assay does not meet the WHO-suggested minimal requirement for a non-sputum near point-of-care target product profile assay, and should not be used to diagnose active tuberculosis.^13^^,^^36^

For immunocompetent individuals who did not receive TPT, a positive QFT+ result was associated with a 14-fold higher incidence of active tuberculosis compared to individuals with negative QFT+ results, closely resembling the IRR of 10.1 found among contacts of persons with active tuberculosis in a prospective cohort study from the UK.^37^ Among immunocompromised individuals, the incidence of active tuberculosis was sevenfold higher in those with positive QFT+ results compared to immunocompetent controls with negative QFT+ results. In line with our previous study on the performance of two different IGRA tests including the previous QuantiFERON-TB Gold in-tube test,^16^ incident cases of tuberculosis occurred almost exclusively in PLHIV. We also show that the incidence of tuberculosis was 18-fold higher among immunocompromised individuals with indeterminate QFT+ results compared to immunocompetent individuals with negative QFT+, similar in magnitude to the IRR of 18.7 observed among foreign-born Norwegians with indeterminate versus negative QuantiFERON-TB Gold in-tube results.^38^ None of the 109 individuals who took TPT developed active tuberculosis. However, only 32/213 (15.0%) immunocompromised individuals with a positive QFT+ result received TPT as recommended by WHO guidelines (12.5% (14/112) PLHIV, and 17.8% (18/101) HIV-seronegative immunocompromised persons).

Among PLHIV, all patients developing tuberculosis originated from medium- or high-incidence counties of tuberculosis (Brazil, Colombia and Republic of Moldova) and were late presenters in line with a previous study on PLHIV with delayed initiation of ART.^39^ In this population, all cases of incident tuberculosis occurred among PLHIV with detectable HIV-replication, with incidence rates being 216- and 44-fold higher among those with indeterminate and positive QFT+ results, respectively, compared to immunocompetent controls with negative QFT+ results. ART and control of HIV-replication appear to be important, as none of 455 PLHIV on ART with undetectable HIV-load developed tuberculosis during 1413 years of follow-up, although 71 had positive QFT+ results at the time of screening. Recent data from >270.000 PLHIV with almost 2-million person-years of follow-up showed a substantial CD4-count-independent association of HIV-load with the risk for tuberculosis in PLHIV. As in our study, there was a higher incidence of tuberculosis among people born in tuberculosis endemic countries.^40^ Current WHO guidelines suggest TPT for all PLHIV when active tuberculosis has been excluded, no contraindications exist, and exposure to tuberculosis is likely.^6^ WHO also recommends skin- or IGRA testing for other immunocompromised individuals and offering TPT for those with a positive (or an unavailable) test result in the absence of contraindications. However, many physicians participating in our study apparently found it difficult to adhere to WHO guidelines for tuberculosis prevention. It should be highlighted that PLHIV, especially those from high-burden tuberculosis countries with a reactive IGRA or TST should be monitored carefully during follow up.^41^ Our study results suggest that a stratified approach may be more reasonable in countries of low tuberculosis incidence, based on the high risk for progression to tuberculosis in PLHIV with detectable HIV-replication and/or low CD4-counts, and a low risk in immunocompromised individuals with no additional risk factors for tuberculosis.

Our study has limitations. First, while we enrolled a large number of immunocompromised individuals from five different groups of immunocompromised individuals, the results must be interpreted with caution and sample sizes in the individual groups are not sufficiently large for definitive conclusions, especially in some subgroups of immunocompromised individuals such as those receiving treatment with TNF-antagonists and JAK-inhibitors, or stem cell transplant recipients. Second, our patient and control groups were not matched for risk factors of prior exposure and the overall number of individuals developing tuberculosis was low. Moreover, the study was not sufficiently powered to identify regional differences in the risk of active tuberculosis in immunocompromised individuals in countries of Europe with a higher tuberculosis incidence. Third, although we believe confounding by indication did not appreciably impact our overall results due to the rather very low use of TPT in our study population, we acknowledge that this bias may have been present, potentially underestimating the ability of QFT testing to predict new tuberculosis cases. Finally, the study was not designed to provide precise data on timing of HIV testing, choice of and adherence to antiretroviral therapy among PLHIV.

In conclusion, this study found that the risk of progression to active tuberculosis among immunocompromised individuals in countries with low tuberculosis incidence was low over a median follow-up of two years. The predictive value of the QFT+ test for identifying individuals who would develop active tuberculosis appeared limited in this setting, with the possible exception of PLHIV who had ongoing viral replication and low CD4 counts. Furthermore, the TB2 tube did not show a clear advantage over the TB1 tube in predicting progression to active tuberculosis. Nevertheless, there are still no alternative tests for IGRAs or TST with better predictive values for progression or with better ability to distinguish active and non-active stages outside of clinical research.^42^, ^43^, ^44^ This low predictive value and the need to consider additional risk factors is also reflected by our observations of substantial deviations from international TPT guidelines, with both under- and over-treatment observed. Our results underscore the need for a nuanced approach to tuberculosis prevention in immunocompromised individuals, potentially incorporating additional risk stratification beyond QFT+ results alone. However, continuing evaluation of the role of immunodiagnostic testing for tuberculosis prevention strategies in immunocompromised individuals is warranted to inform policy in diverse clinical contexts.

## Contributors

MS made a substantial contribution to the conception and design of the work, to the acquisition, analysis and interpretation of data for the work, accessed and verified the data, wrote the manuscript, critically revised the manuscript for important intellectual content, and gave final approval of the current version to be published. FvL made a substantial contribution to the conception and design of the work, to the data analysis plan, and to the interpretation of data for the work, had access to the data, critically revised the manuscript for important intellectual content, and gave final approval of the current version to be published. LM and OSP made a substantial contribution to the interpretation of data for the work, accessed and verified the data, performed statistical analysis, wrote the manuscript, critically revised the manuscript for important intellectual content, and gave final approval of the current version to be published. CL made a substantial contribution to the conception and design of the work, to the acquisition, analysis and interpretation of data for the work, had access to the data, wrote the manuscript, critically revised the manuscript for important intellectual content, and gave final approval of the current version to be published. All other authors made a contribution to the acquisition of the data for the work, had access to the data, critically revised the manuscript for important intellectual content, and gave final approval of the current version to be published. All authors agree to be accountable for all aspects of the work in ensuring that questions related to the accuracy or integrity of any part of the work are appropriately investigated and resolved.

## Data sharing statement

Data collected for the study, including individual participant data and a data dictionary defining each field in the set, will be made available to others with investigator support once all relevant substudies are reported and completed. Data can be made available upon reasonable request to the corresponding author after approval of a proposal within the TBnet steering committee.

## Declaration of interests

Aase Bengaard Andersen has a patent regarding ESAT-6 for use in Quantiferon test issued via Statens Serum Institute and sold more than 10 years ago, and is chairman of Data Safety Monitoring Board regarding phase 1 vaccine study sponsored by Statens Serum Institute (nTB-01) finalized in December 2024. Graham Bothamley reports to have been past chair of TBnet. James Brown has received a grant from Asthma + Lung UK for a project investigating serological diagnosis of mycobacterium avium lung disease, paid to the institution. Delia Goletti has received consulting fees by PBD Biotech to participate at a scientific board, and a honorarium by Biomerieux for a presentation. Harald Hoffmann has received consulting fees, expert testimonies, and travel fees by USAID, the German government (KfW and BMZ), the global Fund, UNDP, and GIZ. Barbara Kalsdorf has received honoraria for lectures and/or support for meetings and travel by Insmed Germany GmbH, AstraZeneca, Chiesi, Boehringer Ingelheim, and Grifols. Berit Lange has received grant support by the German BMG and BMBF, has received honoraria from Freiburg University for a presentation, and has received support for attending meetings by Roche, MSD, Janssen Cilag, and Abbott, and declares membership of several boards (Expert Council ‘Health and Resilience’, Federal Chancellery, Standing Vaccination Commission at the Robert Koch Institute, Elected (Deputy) President of the German Society for Epidemiology (DGEpi), deputy in 2023 and 2026, president in 2024 and 2025; Part of the pool of experts of the Federal Ministry of Education and Research (BMBF) for consultations on pandemic preparedness and the overall responsiveness of health research to health crises; Member of the Advisory Board for the Pact for Public Health (Pakt ÖGD), Federal Ministry of Health (BMG); Elected Speaker of the Modelling Network for Severe Infectious Diseases in Germany (MONID); Member of the DZIF Internal Advisory Board; Elected member of the steering committee of TBnet; Member of the Working group “Sounding Board Prioritization List” of the National Vaccination Committee of the Federal Ministry of Social Affairs, Health, Care and Consumer Protection (Austria)). Christoph Lange is supported by the German Center of Infection Research, and has received consulting fees and honoraria from Insmed Germany GmbH, Gilead, AstraZeneca, or GSK, and is a member of the Data Safety Board of trials from Medicines sans Frontiers. Marc Lipman is an unpaid trustee of the NTM Network UK, of NTM patient care UK, and Chair of the UK Joint Tuberculosis Committee. Martin Nitschke has received honoraria for lectures by AstraZeneca, Boehringer Ingelheim, travel support by Lilly, and participated in Data Safety Monitoring Boards for AstraZeneca and Boehringer Ingelheim. Pernille Ravn has received honoraria by Takeda for lectures, and has a patent on IP-10 as TB diagnostic agent, and has received Quantiferon kits from SSI Diagnostics for research purposes. Martina Sester and TBnet have received QuantiFERON kits for the present study free of charge. Martina Sester has received grant support by Astellas, Biotest, and Takeda to the institution Saarland University outside of the submitted work, and has received honoraria for presentations or work in Data Safety monitoring Boards, and travel support for meetings by Biotest, Novartis, Takeda, MSD, and Moderna. Dirk Wagner has participated in an advisory board by Pfizer on NTM-PD. All other authors do not have any conflict of interest to declare.
