# Correction to Diagnostic accuracy and predictive value of the QuantiFERON-TB gold plus assay for tuberculosis in immunocompromised individuals: a prospective TBnet study Lancet Reg Health Eur 57 (2025) 101416 LLRHEUROPE-D-25-00310

**DOI:** 10.1016/j.lanepe.2025.101523

**Published:** 2025-11-26

**Authors:** Martina Sester, Neus Altet-Gomez, Åse Bengaard Andersen, Miguel Arias-Guillén, Korkut Avsar, Anne-Marte Bakken Kran, Graham Bothamley, Anne Christine Nordholm Breschel, James Brown, Dumitru Chesov, Nelly Ciobanu, Daniela Maria Cirillo, Valeriu Crudu, Malu de Souza Galvao, Asli Görek Dilektasli, José Dominguez, Raquel Duarte, Anne Ma Dyrhol-Riise, Delia Goletti, Harald Hoffmann, Elmira Ibraim, Barbara Kalsdorf, Marcin Krawczyk, Heinke Kunst, Berit Lange, Marc Lipman, Alberto Matteelli, Piotr Milkiewicz, David Neyer, Martin Nitschke, Haluk Barbaros Oral, Juan José Palacios-Gutiérrez, Elisa Petruccioli, Joanna Raszeja-Wyszomirska, Pernille Ravn, Jan Rupp, Hanna-Elisa Spohn, Corina Toader, Raquel Villar-Hernandez, Dirk Wagner, Frank van Leth, Leonardo Martinez, Ole Skouvig Pedersen, Christoph Lange

**Affiliations:** a Department of Transplant and Infection Immunology, Saarland University, Homburg, Germany; b Center for Gender-specific Biology and Medicine (CGBM), Saarland University, Homburg, Germany; c Unidad Clinica de Tratamiento. Directamente Observado “Serveis Clinics”, Barcelona, Spain; d Department of Infectious Diseases, Odense University Hospital, Odense, Denmark; e Respiratory Department, Central University Hospital of Asturias, ISPA, Faculty of Medicine, University of Oviedo, CIBER-Respiratory Diseases, Carlos III Health Institute, Oviedo, Spain; f Department of Infectious Diseases, Asklepios Fachklinik München-Gauting, Munich, Germany; g Division of Infection Control, Norwegian Institute of Public Health (NIPH), Oslo, Norway; h Homerton University Hospital, London, UK; i Department of Infectious Disease Epidemiology and Prevention, Statens Serum Institut, Copenhagen, Denmark; j Department of Infectious Diseases, Rigshospitalet, Copenhagen University Hospital, Copenhagen, Denmark; k Department of Respiratory Medicine, Royal Free London NHS Trust and UCL Respiratory, University College London, UK; l Discipline of Pneumology and Allergology Nicolae Testemitanu State University of Medicine and Pharmacy, Republic of Moldova; m Clinical Infectious Disease. Department of Clinical Infectious Diseases, Research Center Borstel, Leibniz Lung Center, Borstel, Germany; n German Center for Infection Research (DZIF), Tuberculosis Unit, Borstel, Germany; o National TB Reference Laboratory, Pneumology Institute, Chisinau, Moldova; p IRCCS San Raffaele Scientific Institute, Milan, Italy; q Pneumology Service, Hospital Universitari Vall d’Hebron, Barcelona, Spain; r Department of Pulmonary Medicine, Bursa Uludag University Faculty of Medicine, Bursa, Turkey; s Servei de Microbiologia, Hospital Universitari Germans Trias i Pujol, Institut d’Investigació Germans Trias i Pujol, Badalona, Spain; t CIBER Enfermedades Respiratorias, Badalona, Spain; u Universidad Autónoma de Barcelona, Barcelona, Spain; v Instituto Nacional De Saúde Dr Ricardo Jorge do Porto, Porto, Portugal; w Instituto de Saúde Pública da Universidade do Porto; Porto, Portugal; x Department of Infectious Diseases, Oslo University Hospital, Oslo, Norway; y Institute of Clinical Medicine, University of Oslo, Oslo, Norway; z Translational Research Unit, National Institute for Infectious Diseases “Lazzaro Spallanzani”, Istituto di Ricovero e Cura a Carattere Scientifico (IRCCS), Rome, Italy; aa Institute of Microbiology and Laboratory Medicine, IML red GmbH; WHO - Supranational Tuberculosis Reference Laboratory Munich-Gauting; Gauting, Germany; ab SYNLAB Gauting, SYNLAB MVZ Dachau GmbH, Munich-Gauting, Germany; ac Department of Clinical Research, Marius Nasta Institute of Pneumophtiziology, Bucharest, Romania; ad Respiratory Medicine & International Health, University of Lübeck, Lübeck, Germany; ae Department of Gastroenterology, Hepatology and Transplant Medicine, Medical Faculty, University of Duisburg-Essen, Essen, Germany; af Laboratory of Metabolic Liver Diseases, Department of General, Transplant and Liver Surgery, Centre for Preclinical Research, Medical University of Warsaw, Warsaw, Poland; ag Queen Mary & Barts Health Tuberculosis Centre, Blizard Institute, Faculty of Medicine & Dentistry, Queen Mary University of London, London, UK; ah Department of Epidemiology, Helmholtz Centre for Infection Research (HZI), Braunschweig, Germany; ai German Center for Infection Research (DZIF), Braunschweig, Germany; aj Clinic of Infectious and Tropical Diseases, Department of Clinical and Experimental Medicine, WHO Collaboration Centre for Tuberculosis Prevention, University of Brescia, Brescia, Italy; ak Department of Hepatology, Transplantology and Internal Medicine, Medical University of Warsaw, Warsaw, Poland; al Translational Medicine Group, Pomeranian Medical University, Szczecin, Poland; am Division of Infectious Diseases, Department of Internal Medicine II, Freiburg University Medical Centre, Freiburg, Germany; an Transplant Center, University Hospital of Schleswig-Holstein, Lübeck, Germany; ao Department of Immunology, Faculty of Medicine, Bursa Uludag University, Bursa, Turkey; ap Regional Mycobacteria Reference Unit, Central University Hospital of Asturias, Instituto de Investigación Sanitaria del Principado de Asturias (ISPA), Oviedo, Spain; aq Section of Infectious Diseases, Department of Medicine, Herlev and Gentofte Hospital, University of Copenhagen, Denmark; ar Infectious Disease Clinic and Institute of Medical Microbiology, University Hospital Schleswig-Holstein, Lübeck, Germany; as German Center for Infection Research (DZIF), Partner Site Hamburg-Lübeck-Borstel-Riems, Lübeck, Germany; at Pathology Department, Marius Nasta Institute of Pneumophysiology, Bucharest, Romania; au Department of Health Sciences, Vrij Universiteit Amsterdam, the Netherlands; av Amsterdam Public Health Research Institute, Amsterdam, the Netherlands; aw Boston University, School of Public Health, Department of Epidemiology, Boston, MA, USA; ax Department of Respiratory Diseases and Allergy, Aarhus University Hospital, Aarhus, Denmark; ay Department of Clinical Medicine, Aarhus University, Aarhus, Denmark; az Global TB Program, Baylor College of Medicine and Texas Children's Hospital, Houston, TX, USA; ba Queen Mary University of London, London, UK; bb London School of Hygiene and Tropical Medicine, London, UK

**Corrected text**

The assignment of the institutions for two authors (Elisa Petruccioli and Christoph Lange) were not correct.

The correct affiliations are as follows:

Elisa Petruccioli^z^

^z^Translational Research Unit, National Institute for Infectious Diseases “Lazzaro Spallanzani”, Istituto di Ricovero e Cura a Carattere Scientifico (IRCCS), Rome, Italy.

Christoph Lange^m, n, ad, az, bd^

^m^Clinical Infectious Disease. Department of Clinical Infectious Diseases, Research Center Borstel, Leibniz Lung Center, Borstel, Germany

^n^German Center for Infection Research (DZIF), Tuberculosis Unit, Borstel, Germany;

^ad^Respiratory Medicine & International Health, University of Lübeck, Lübeck, Germany;

^az^Global TB Program, Baylor College of Medicine and Texas Childrenś Hospital, Houston, TX, USA.

^bd^equal contribution

