# Supplementary material for: Diagnostic accuracy and predictive value of the QuantiFERON-TB gold plus assay for tuberculosis in immunocompromised individuals: a prospective TBnet study
Source: Lancet Reg Health Eur. 2025 Aug 6;57:101416. doi: 10.1016/j.lanepe.2025.101416 (PMC12355092; doi:10.1016/j.lanepe.2025.101416)
Supplement: Supplementary Material [file mmc1.docx]

**Supplementary Information**

**Diagnostic accuracy and predictive value of the QuantiFERON-TB Gold Plus assay for tuberculosis in immunocompromised individuals: A prospective TBnet study**

**Content**

[Supplementary methods 3](#_Toc199604948)

[Supplementary tables 4](#_Toc199604949)

[Supplementary Table S1: Standards for Reporting Diagnostic accuracy studies checklist 4](#_Toc199604950)

[Supplementary Table S2: Immunosuppressive drugs in persons with rheumatoid arthritis. 6](#_Toc199604951)

[Supplementary Table S3: Immunosuppressive drugs in persons after solid organ and stem cell transplantation. 7](#_Toc199604952)

[Supplementary Table S4: Sensitivity and specificity of the QFT+ result stratified by status of immunosuppression 8](#_Toc199604953)

[Supplementary Table S5: Incidence of tuberculosis for PLHIV without tuberculosis preventive therapy stratified by QFT+ result and HIV-load 9](#_Toc199604954)

[Supplementary Table S6A-F: Individuals at risk based on Aalen-Johansen calculation referring to figure 3a-f. 10](#_Toc199604955)

[Supplementary Figures 12](#_Toc199604956)

[Supplementary Figure S1 12](#_Toc199604957)

[Supplementary figure S2 14](#_Toc199604958)

[References for supplementary information 16](#_Toc199604959)

# Supplementary methods

The QFT+ assay was performed according to the manufacturer’s instructions (Qiagen, Hilden, Germany). Briefly, 1ml-volumes of heparinized whole blood were incubated in two different tubes encompassing different *M. tuberculosis*-specific peptide cocktails. The TB1-tube contains peptides derived from ESAT-6 and CFP-10 (as in the previously used tube of the QuantiFERON-TB Gold in tube assay except for peptides derived from TB7.7) primarily inducing cytokine release from CD4 T cells. The TB2-tube contains both peptides from TB1 and CFP-10 derived peptides optimized to stimulate CD8 T cells.^[1](#_ENREF_1),[2](#_ENREF_2)^ Moreover, negative and the positive control tubes were included (Nil and mitogen phytohemagglutinine (PHA), respectively). All samples were stimulated at the participating sites for 16-24 hours within 16 hours after venepuncture, and IFN-γ levels secreted into the supernatants were quantified using an enzyme-linked immunosorbant assay (ELISA). Both quantitative test results (IFN-γ in IU/ml) and qualitative test results (positive, negative, indeterminate) as per manufacturer’s instructions and for each tube were recorded. The QFT+ test was scored positive if the IFN-γ levels in either the TB1. or the TB2-tube or both tubes were (≥0.35 IU/ml) after subtraction of the Nil control. The upper limit of quantification for IFN-γ levels as detected by the ELISA was 10 IU/ml.

# Supplementary tables

## Supplementary Table S1: Standards for Reporting Diagnostic accuracy studies checklist

|  | **Section & Topic** | **No** | **Item** | **Reported on page #** |
| --- | --- | --- | --- | --- |
|  |  |  |  |  |
|  | **TITLE OR ABSTRACT** |  |  |  |
|  |  | **1** | Identification as a study of diagnostic accuracy using at least one measure of accuracy (such as sensitivity, specificity, predictive values, or AUC) | 1 |
|  | **ABSTRACT** |  |  |  |
|  |  | **2** | Structured summary of study design, methods, results, and conclusions (for specific guidance, see STARD for Abstracts) | pp. 7 and 8 |
|  | **INTRODUCTION** |  |  |  |
|  |  | **3** | Scientific and clinical background, including the intended use and clinical role of the index test | Research in context box + pp. 12-14 |
|  |  | **4** | Study objectives and hypotheses | 14 |
|  | **METHODS** |  |  |  |
|  | *Study design* | **5** | Whether data collection was planned before the index test and reference standard were performed (prospective study) or after (retrospective study) | Title + p. 15 |
|  | *Participants* | **6** | Eligibility criteria | 15-16 |
|  |  | **7** | On what basis potentially eligible participants were identified  (such as symptoms, results from previous tests, inclusion in registry) | Missing |
|  |  | **8** | Where and when potentially eligible participants were identified (setting, location and dates) | 15 |
|  |  | **9** | Whether participants formed a consecutive, random or convenience series | 15 |
|  | *Test methods* | **10a** | Index test, in sufficient detail to allow replication | 17 and supplementary methods |
|  |  | **10b** | Reference standard, in sufficient detail to allow replication | 17 |
|  |  | **11** | Rationale for choosing the reference standard (if alternatives exist) | 17 |
|  |  | **12a** | Definition of and rationale for test positivity cut-offs or result categories  of the index test, distinguishing pre-specified from exploratory | 17 and supplementary methods |
|  |  | **12b** | Definition of and rationale for test positivity cut-offs or result categories  of the reference standard, distinguishing pre-specified from exploratory | NA |
|  |  | **13a** | Whether clinical information and reference standard results were available to the performers/readers of the index test | 17 |
|  |  | **13b** | Whether clinical information and index test results were available to the assessors of the reference standard | NA |
|  | *Analysis* | **14** | Methods for estimating or comparing measures of diagnostic accuracy | 17-19 |
|  |  | **15** | How indeterminate index test or reference standard results were handled | 17-19 |
|  |  | **16** | How missing data on the index test and reference standard were handled | 17-19 |
|  |  | **17** | Any analyses of variability in diagnostic accuracy, distinguishing pre-specified from exploratory | 17-19 |
|  |  | **18** | Intended sample size and how it was determined | Missing |
|  | **RESULTS** |  |  |  |
|  | *Participants* | **19** | Flow of participants, using a diagram | Figure 2a |
|  |  | **20** | Baseline demographic and clinical characteristics of participants | Table 1 |
|  |  | **21a** | Distribution of severity of disease in those with the target condition | Table 1 and 2 |
|  |  | **21b** | Distribution of alternative diagnoses in those without the target condition | NA |
|  |  | **22** | Time interval and any clinical interventions between index test and reference standard | Figure 2a |
|  | *Test results* | **23** | Cross tabulation of the index test results (or their distribution)  by the results of the reference standard | Supplementary table S1 |
|  |  | **24** | Estimates of diagnostic accuracy and their precision (such as 95% confidence intervals) | Supplementary table S1, figure S2, figure S3, p. 22 |
|  |  | **25** | Any adverse events from performing the index test or the reference standard | NA |
|  | **DISCUSSION** |  |  |  |
|  |  | **26** | Study limitations, including sources of potential bias, statistical uncertainty, and generalisability | 29 |
|  |  | **27** | Implications for practice, including the intended use and clinical role of the index test | 29-30 |
|  | **OTHER INFORMATION** |  |  |  |
|  |  | **28** | Registration number and name of registry | 7 |
|  |  | **29** | Where the full study protocol can be accessed | 7 |
|  |  | **30** | Sources of funding and other support; role of funders | 5, 8, 19 |
|  |  |  |  |  |

## Supplementary Table S2: Immunosuppressive drugs in persons with rheumatoid arthritis.

| **n (%)** | **Rheumatoid arthritis n=292** | |
| --- | --- | --- |
| Number of drugs |  |  |
| <3 drugs | 240 (82.2%) | |
| ≥3 drugs | 52 (17.8%) | |
| Type of drug regimen | no steroid | steroid |
| no additional drug | 16 (5.5%) | 21 (7.2%) |
| csDMARD | 57 (19.5%) | 71 (24.3%) |
| bDMARD (TNF antagonist) | 15 (5.1%) | 12 (4.1%) |
| cDMARD+bDMARD (TNF antagonist) | 30 (10.3%) | 14 (4.8%) |
| b/tsDMARD (others) | 12 (4.1%) | 8 (2.7%) |
| cDMARD+bDMARD (others) | 10 (3.4%) | 13 (4.5%) |
| bDMARD (TNF antagonist)+bDMARD (other) | 3 (1.0%) | 2 (0.7%) |
| cDMARD+ bDMARD (TNF antagonist)+bDMARD (other) | 3 (1.0%) | 5 (1.7%) |

DMARD, disease modifying antirheumatic drug; csDMARD, conventional DMARD (methotrexate, hydrochloroquine, sulfasalazine, leflunomide, mycophenolate mofetile, azathioprine); bDMARD, biological DMARD (TNF antagonist): adalimumab, infliximab, certolizumab, golimumab, eternacept); b/tsDMARD biological or targeted synthetic DMARDS (others): abatacept, anakinra, apremilast, belimumab, rituximab, secukinumab, tocilizumab, tofacitinib).

## Supplementary Table S3: Immunosuppressive drugs in persons after solid organ and stem cell transplantation.

| **n (%)** | **Solid organ transplantation  n=239** | **Stem cell transplantation n=193** |
| --- | --- | --- |
| Time after transplantation |  |  |
| ≤1 year | 77 (32.2%) | 40 (20.7%) |
| >1 year | 162 (67.8%) | 153 (79.3%) |
| Drug score* |  |  |
| Low (<1) | 16 (6.7%) | 146 (75.6%) |
| Medium (≥1-2.5) | 124 (51.9%) | 39 (20.2%) |
| High (≥2.5) | 99 (41.4%) | 8 (4.1%) |

*drug score was calculated as described before[^3^](#_ENREF_3) as a composite score based on the number of immunosuppressive drugs and their dosage.

## Supplementary Table S4: Sensitivity and specificity of the QFT+ result stratified by status of immunosuppression

|  | **Immunocompetent** | **Immunocompromised** |
| --- | --- | --- |
|  | n=530 | n=1,201 |
| Sensitivity (95% CI)^*^ | 81.4% (76.6-85.3%) (249/306) | 70.0% (52.1-83.3%) (21/30) |
| Specificity (95% CI)^$^ | 96.0% (92.5-97.9%)  (215/224) | 91.4% (89.6-92.9%)  (1,070/1,171) |

^*^Proportion of positive test results in participants with active tuberculosis; ^$^Proportion of negative test results in participants without known risk factors or a history of *M. tuberculosis* exposure (224 low-risk controls and 1171 immunocompromised persons, see also table 1); CI, confidence interval.

## **Supplementary Table S5: Incidence of tuberculosis for PLHIV without tuberculosis preventive therapy stratified by QFT+ result and HIV-load**

|  | **Test-result^§^** | **HIV-load [copies/ml]** | **n** | **PY at risk** | **TB cases** | **Incidence*** | **Incidence rate ratio^†^** |
| --- | --- | --- | --- | --- | --- | --- | --- |
| PLHIV | Negative | <50 | 457 | 1413 | 0 | 0 | - |
|  | Indeterminate | <50 | 3 | 6 | 0 | 0 | - |
|  | Positive | <50 | 68 | 162 | 0 | 0 | - |
|  | Negative | ≥50 | 87 | 232 | 0 | 0 | - |
|  | Indeterminate | ≥50 | 2 | 5 | 1 | 20.0 (2.8-142-0) | 216.0 (13.5-3446.0) |
|  | Positive | ≥50 | 30 | 75 | 3 | 4.1 (1.3-12.4) | 44.3 (4.6-426.0) |

*Incidence is given per 100 person-years (PY); the rates refer to the cumulative rates after testing for *M. tuberculosis* infection. ^§^This analysis includes all PLHIV with known HIV-load without preventive therapy, stratified by positive, negative or indeterminate QFT+ results. **^†^**The reference groups comprised immunocompetent individuals with negative QFT+ results without preventive therapy; PY, person-years; PLHIV, people living with HIV; TB, tuberculosis.

## Supplementary Table S6A-F: Individuals at risk based on Aalen-Johansen calculation referring to figure 3a-f.

| **Fig. 3a** | **Months** | **0** | **10** | **20** | **30** | **40** | **50** | **60** |
| --- | --- | --- | --- | --- | --- | --- | --- | --- |
| **At risk** | QFT+ negative | 1706 | 1605 | 1496 | 963 | 551 | 256 | 5 |
|  | QFT+ positive | 237 | 219 | 208 | 119 | 43 | 19 | 0 |

| **Fig. 3b** | **Months** | **0** | **10** | **20** | **30** | **40** | **50** | **60** |
| --- | --- | --- | --- | --- | --- | --- | --- | --- |
| **At risk** | QFT+ negative, HIV-negative | 1156 | 1072 | 994 | 677 | 381 | 99 | 2 |
|  | QFT+ negative, HIV-positive | 550 | 533 | 502 | 286 | 170 | 157 | 3 |
|  | QFT+ positive, HIV-negative | 139 | 124 | 120 | 88 | 37 | 17 | 0 |
|  | QFT+ positive, HIV-positive | 98 | 95 | 88 | 31 | 6 | 2 | 0 |

| **Fig. 3c** | **Months** | **0** | **10** | **20** | **30** | **40** | **50** | **60** |
| --- | --- | --- | --- | --- | --- | --- | --- | --- |
| **At risk** | HIV load < 50 copies/mL | 528 | 515 | 480 | 252 | 165 | 151 | 3 |
|  | HIV load ≥ 50 copies/mL | 119 | 112 | 109 | 62 | 11 | 8 | 0 |

Continued on next page.

| **Fig. 3d** | **Months** | | **0** | | **10** | | **20** | | **30** | | **40** | | **50** | **60** |
| --- | --- | --- | --- | --- | --- | --- | --- | --- | --- | --- | --- | --- | --- | --- |
| **At risk** | HIV load < 50 copies/mL, CD4 count ≥ 200 cells/mL | 495 | | 483 | | 450 | | 239 | | 156 | | 143 | | 3 |
|  | HIV load < 50 copies/mL, CD4 count ≥ 200 cells/mL | 33 | | 32 | | 30 | | 13 | | 9 | | 8 | | 0 |
|  | HIV load ≥ 50 copies/mL, CD4 count < 200 cells/mL | 101 | | 97 | | 96 | | 55 | | 10 | | 7 | | 0 |
|  | HIV load ≥ 50 copies/mL, CD4 count < 200 cells/mL | 18 | | 15 | | 13 | | 7 | | 1 | | 1 | | 0 |

| **Fig. 3e** | **Months** | **0** | **10** | **20** | **30** | **40** | **50** | **60** |
| --- | --- | --- | --- | --- | --- | --- | --- | --- |
| **At risk** | HIV load < 50 copies/mL, QFT+ negative | 457 | 444 | 415 | 236 | 161 | 150 | 3 |
|  | HIV load < 50 copies/mL, QFT+ positive | 68 | 68 | 62 | 16 | 4 | 1 | 0 |
|  | HIV load ≥ 50 copies/mL, QFT+ negative | 87 | 83 | 81 | 46 | 9 | 7 | 0 |
|  | HIV load ≥ 50 copies/mL, QFT+ positive | 30 | 27 | 26 | 15 | 2 | 1 | 0 |

| **Fig. 3f** | **Months** | **0** | **10** | **20** | **30** | **40** | **50** | **60** |
| --- | --- | --- | --- | --- | --- | --- | --- | --- |
| **At risk** | HIV load < 50 copies/mL, high incidence | 182 | 180 | 166 | 34 | 14 | 14 | 1 |
|  | HIV load < 50 copies/mL, low incidence | 344 | 333 | 312 | 217 | 151 | 137 | 2 |
|  | HIV load ≥ 50 copies/mL, high incidence | 52 | 50 | 48 | 15 | 2 | 1 | 0 |
|  | HIV load ≥ 50 copies/mL, low incidence | 67 | 62 | 61 | 47 | 9 | 7 | 0 |

# Supplementary Figures

## Supplementary Figure S1

| **a** | 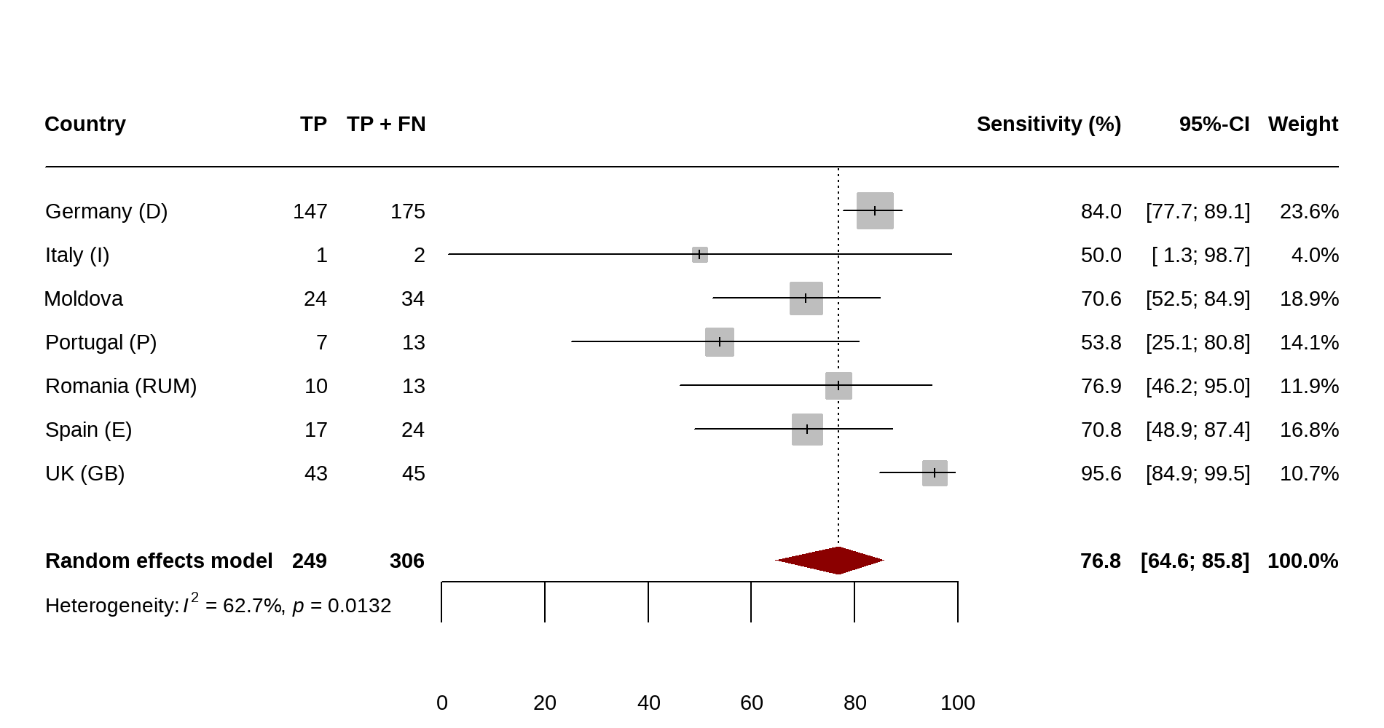 |
| --- | --- |
| **b** | 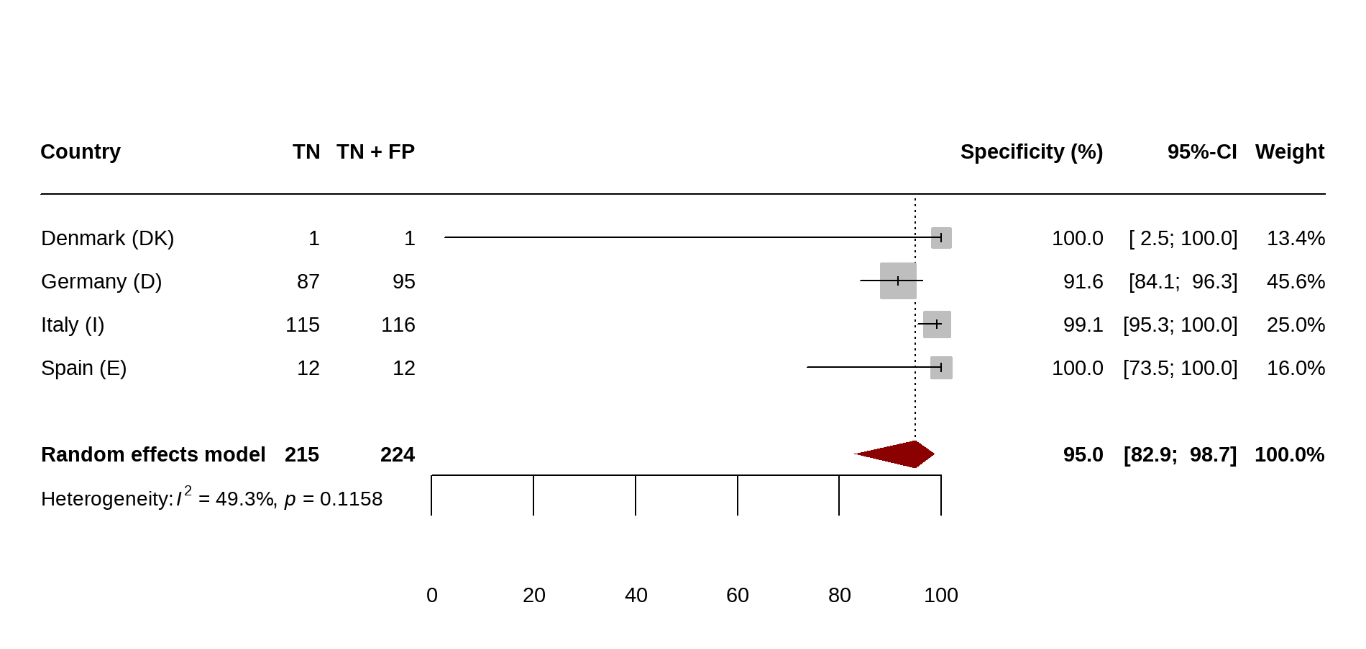 |

**Supplementary Figure S1: Pooled sensitivity and specificity of the QFT+ test for detection of active tuberculosis in immunocompetent persons.** Pooled estimates for **(a)** sensitivity and **(b)** specificity were calculated using a random-effects meta-analysis of proportions with logit transformation. Between-country variability was estimated using the DerSimonian–Laird method. The resulting tau-squared value was 0.4 (95% CI 0.0-3.6) for sensitivities and 0.9 (95% CI 0.0-27.7) for specificities. Country weights were assigned using the inverse variance method. Confidence intervals for individual studies were calculated using the Clopper–Pearson method. Knapp–Hartung adjustments were applied to the random-effects model. Proportions are presented on the original scale (%). TP, true positive; FN, false negative; 95% CI, 95% confidence interval.

## Supplementary figure S2

| **a** | 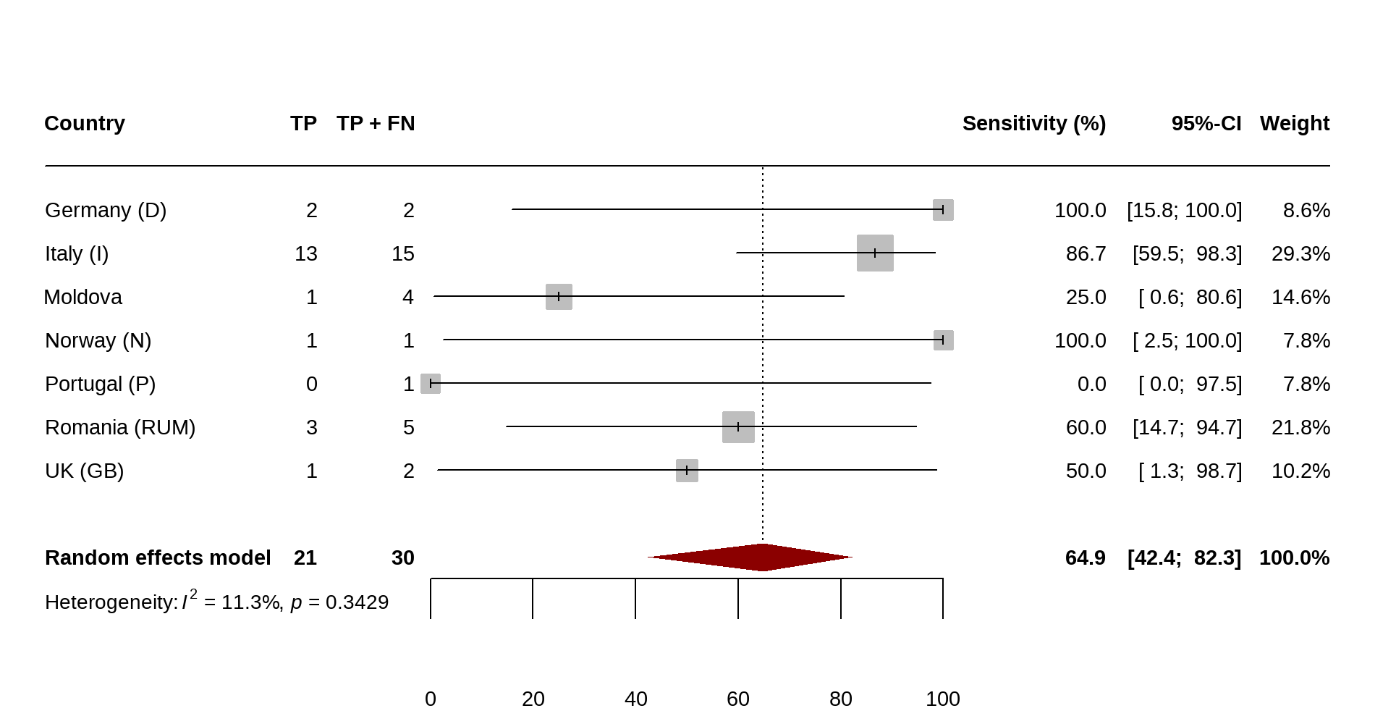 |
| --- | --- |
| **b** | 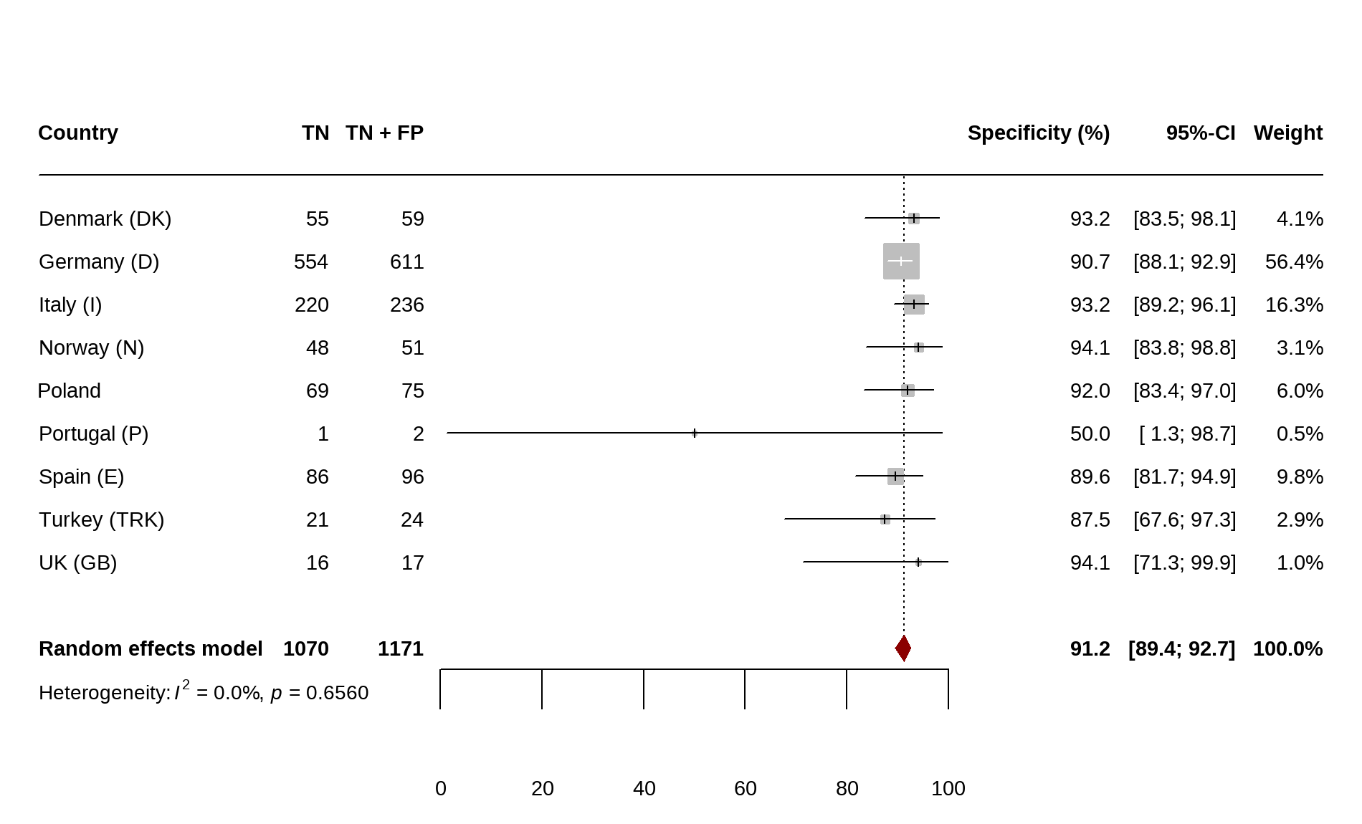 |

**Supplementary Figure S2: Pooled sensitivity and specificity of the QFT+ test for detection of active tuberculosis in immunocompromised patients.** Pooled estimates for **(a)** sensitivity and **(b)** specificity were calculated using a random-effects meta-analysis of proportions with logit transformation. Between-country variability was estimated using the DerSimonian–Laird method. The resulting tau-squared value was 0.2 (95% CI 0.0-5.8) for sensitivities and 0.0 (95% CI 0.0-0.8) for specificities. Country weights were assigned using the inverse variance method. Confidence intervals for individual studies were calculated using the Clopper–Pearson method. Knapp–Hartung adjustments were applied to the random-effects model. Proportions are presented on the original scale (%). TP, true positive; FN, false negative; 95% CI, 95% confidence interval.

# References for supplementary information

1. Barcellini L, Borroni E, Brown J, et al. First independent evaluation of QuantiFERON-TB Plus performance. *Eur Respir J* 2016; **47**(5): 1587-90.

2. Allen NP, Swarbrick G, Cansler M, et al. Characterization of specific CD4 and CD8 T-cell responses in QuantiFERON TB Gold-Plus TB1 and TB2 tubes. *Tuberculosis (Edinb)* 2018; **113**: 239-41.

3. Sester M, van Leth F, Bruchfeld J, et al. Risk assessment of tuberculosis in immunocompromised patients. A TBNET study. *Am J Respir Crit Care Med* 2014; **190**(10): 1168-76.
